# Interprofessional simulated learning: short-term associations between simulation and interprofessional collaboration

**DOI:** 10.1186/1741-7015-9-29

**Published:** 2011-03-28

**Authors:** Chris Kenaszchuk, Kathleen MacMillan, Mary van Soeren, Scott Reeves

**Affiliations:** 1 Li Ka Shing Knowledge Institute of St. Michael's Hospital, 30 Bond Street, Toronto, Ontario, M5B 1W8, Canada; 2 School of Health Sciences, Humber Institute of Technology and Advanced Learning, Toronto, Canada; 3 Canadian Health Care Innovations, Guelph, Ontario, Canada; 4 Centre for Faculty Development, Li Ka Shing International Healthcare Education Centre, Toronto, Canada; 5 Wilson Centre for Research in Education, University of Toronto, Canada; 6 Department of Psychiatry, University of Toronto, Canada

## Abstract

**Background:**

Health professions education programs use simulation for teaching and maintaining clinical procedural skills. Simulated learning activities are also becoming useful methods of instruction for interprofessional education. The simulation environment for interprofessional training allows participants to explore collaborative ways of improving communicative aspects of clinical care. Simulation has shown communication improvement within and between health care professions, but the impacts of teamwork simulation on perceptions of others' interprofessional practices and one's own attitudes toward teamwork are largely unknown.

**Methods:**

A single-arm intervention study tested the association between simulated team practice and measures of interprofessional collaboration, nurse-physician relationships, and attitudes toward health care teams. Participants were 154 post-licensure nurses, allied health professionals, and physicians. Self- and proxy-report survey measurements were taken before simulation training and two and six weeks after.

**Results:**

Multilevel modeling revealed little change over the study period. Variation in interprofessional collaboration and attitudes was largely attributable to between-person characteristics. A constructed categorical variable indexing 'leadership capacity' found that participants with highest and lowest values were more likely to endorse shared team leadership over physician centrality.

**Conclusion:**

Results from this study indicate that focusing interprofessional simulation education on shared leadership may provide the most leverage to improve interprofessional care.

## Background

Collaboration, communication, and coordination of care are limited in traditional health care structures by isolated health provider education, regulations under which teams practice, and the historic hierarchy in hospital settings [1-3]. Professionalism creates turf issues [3,4] and socialization by separate education leads to communication gaps across professions [5]. Against this background of health care tradition, some new initiatives have shown interprofessional care to contribute to improved staff morale, greater patient satisfaction and patient safety, and reduced duplication of effort [6]. Hogg et al. [7] reported improved patient outcomes when health professionals worked in a collaborative manner. In primary care settings, patients with mental health and other chronic disease challenges experienced health management improvements when teams were involved [8]. Yet transitioning professionals to work in interprofessional partnerships to achieve a coordinated and participatory approach to decision-making around health and social outcomes has been the unrealized goal of interprofessional care [9].

Interprofessional education (IPE) can yield beneficial outcomes such as improving attitudes, collaborative knowledge, and team functioning [10,11]. Teamwork training should impart knowledge about teamwork and enhance teamwork attitudes [12,13]. Training teams has been integrated into interprofessional simulated learning activities, such as simulated family conferences [14], case based simulations [15], and classroom-based training [16,17]. Studies that have used simulation to promote IPE have reported short-term impacts suggesting improved communication within and between health care professions. The simulation environment provides participants the freedom to make mistakes, correct them and improve communication and processes of care [18]. However, there is limited literature on the effectiveness of simulation education that mimics interprofessional development processes in workshops for practicing health care providers. This is an important gap, because most health care professionals devote little time to non-clinical education.

This study investigated short-term associations between simulation training and two outcomes: health professionals' judgments of interprofessional collaboration (IPC) and teamwork by other groups, and self-reports of attitudes toward working in health care teams. We also examined the relationship between simulation, leadership capacity, and teamwork attitudes because the leadership role is an important concept for effective IPC [3,19].

A college-based school of health sciences in North America developed an eight-hour interprofessional simulated education workshop to train clinical staff in team-based practices. When the workshop began, participants received an introduction to the benefits of interprofessional care and education. Participants were given didactic information and experiential learning opportunities. The workshop content addressed key aspects of developing interprofessional teamwork, such as relational factors (hierarchy and professional power), team processes (routines and rituals), and organizational change (the ability to enact change within the sphere of influence) [[20]:p58]. These activities were intended to demonstrate that attitudes toward interprofessional roles and perspectives affect decision making and consultation, collaboration, and shared leadership [20-22]. A typical workshop then involved participation in two or three different ten-minute simulation activities followed by a thirty-minute group debriefing session with a facilitator. Breaks and meals were provided. At the beginning of each activity, learners received a written description of the scenario outlining their unique role in the simulation. Participants were assigned roles that were different from their real-life professions in order to impart another profession's perspective. Each activity contained roles for family members and different health care professionals. Some included patient roles. The assigned roles reflected the professional groups represented in each workshop to ensure that the facilitated debrief could explore ideas about professional role assumptions and misconceptions. (See Additional File 1: The Gift for an example of a simulation scenario).

## Methods

### Design

This was a single-group, uncontrolled intervention study with longitudinal self- and proxy-report survey data collection. Self-reports are those that have the self as the target of a focal concern. Proxy reports make others the target of a focal concern. Twelve training workshops were conducted in 2008 in group sizes between five and fifteen and were one day in duration. Health care professionals participated in one workshop and were asked to complete self- and proxy-report survey instruments at three times. Time 1 measures were completed on the same day as the simulation session and immediately before its commencement. Time 2 measures were completed approximately 14 days after Time 1 simulation training. Time 3 measures were completed 6 weeks after simulation training. Ethics review boards of the lead and participating institutions approved the study.

### Participants

Participants were licensed, regulated health professionals practicing in a community hospital near Toronto, Canada. Professions represented were physicians, nurses, and other regulated health professionals including dietitians, occupational and physical therapists, pharmacists, social workers, speech-language pathologists, and others. Participants were recruited through information sessions and personal recruitment by an institution-based research assistant. Workshop participation was voluntary. Honoraria were provided to counteract participation barriers due to lost office hours or other work commitments.

### Measures

#### Dependent variables

The Nurses' Opinion Questionnaire (NOQ) [23] taps important dimensions of interprofessional collaboration. In previous work the NOQ was adapted to a new format [24] suitable for use with multiple groups of health professionals. The adapted scale presents a new three-factor structure and writes items to specify 'target' groups appropriate for members of 'rater' groups. Respondents self-identified their profession; we aggregated data to higher-level groups. This was straightforward for nurses and physicians who have common training within their professions. Other professions such as occupational and physical therapists, pharmacists, and social workers were aggregated into a third group, allied health staff. Aggregating created six rater-target combinations: physicians-nurses, physicians-allied health staff, nurses-physicians, nurses-allied health staff, allied health staff-physicians, and allied health staff-nurses. This is a round robin design where respondents are proxy reporters on the collaboration behaviors of group targets. Each group is also a target of two other groups. The adapted scale is called the Interprofessional Collaboration Scale (IPC scale). Four response options were available for the items: strongly disagree (1), disagree (2), agree (3), and strongly agree (4).

The Collegial Nurse-Physician Relations Subscale of the Nursing Work Index (NWI-NPRS) [25] was designed for nurses to report on the quality of nursing work relationships with physicians. The four response options were the same as those for the IPC scale. The IPC and NWI-NPRS items have been provided previously [24].

Three subscales make up the Attitudes Toward Health Care Teams Scale (ATHCTS), measuring self-reported attitudes toward collective teamworking in health care groups [26]. The subscales contain 21 items addressing attitudes toward team value (11 items), attitudes toward team efficiency (5 items), and attitudes toward shared leadership/physician non-centrality (5 items). Items had six response options (disagree/agree × strongly/moderately/somewhat).

#### Independent variable

A composite ordinal-level variable on respondents' leadership capacity was created from three background survey items that had either binary or ordered categorical response options. At the first survey occasion, items requested participants to report whether they provided direct patient care (no/yes), managed any other staff members (no/yes), and the number of years of post-licensure experience they had in the profession (<1, 1-5, 6-10, >10). One point was assigned for each response indicating either no direct patient care, or did manage staff, or had >6 years of post-licensure experience. The variable's range was 0-3 with higher values indicating greater leadership potential.

### Multilevel growth curves

Systematic change and individual differences in change were hypothesized to occur after participation in a training workshop. Between- and within-person variation in the repeated measures taken on IPC scales, the NWI-NPRS, and the ATHCTS were analyzed using multilevel growth curves [27] and estimated with SAS PROC MIXED 9.1 [28]. All models employed an unstructured covariance matrix; were estimated with restricted maximum likelihood; and converged and had admissible variance/covariance components except where noted. Group- and grand-mean centering of variables were not used.

## Results

### Participant characteristics

Participants are described in Tables 1 and 2. Characteristics are presented for Time 1, prior to simulation training. There were 154 health professionals who participated in any of the three survey occasions. Most participants were female, were involved in direct patient care, did not have managerial responsibilities, and had more than 10 years of post-licensure experience in the profession. In each professional group, most participants had one point on leadership capacity and about 20-30% had two or three points; the skewness of the distribution suggests that the construct has face validity. Approximately 75% of physicians, nurses, and allied health professionals were working in primary care or cardiac care units when simulation training began.

**Table 1:** Description of study participants

	Physicians	Nurses	Allied Health Professional
Profession (any participation Time 1-Time 3)	14	96	44

Time 1	13	91	43
Time 2	12	89	41
Time 3	9	86	40

Characteristics *(Time 1)*	***%***	***%***	***%***

Gender			
Female	27.3	98.8	90.5
Male	72.7	1.2	9.5

Direct patient care			
Yes	100.0	89.2	93.0
No	0.0	10.8	7.0

Manage staff			
Yes	45.5	26.0	16.7
No	54.5	74.0	83.3

Experience, yrs			
<1	9.1	4.8	4.8
1-5	27.3	13.2	14.3
6-10	9.1	16.9	38.1
>10	54.5	65.1	42.8

Leadership capacity			
0	9.1	16.9	16.7
1	72.7	54.2	64.3
2	18.2	21.7	16.7
3	0.0	7.2	2.4

Department			
Oncology	0.0	5.0	12.5
Cardiac care	18.2	42.5	40.0
Primary care	54.5	37.5	35.0
Emergency	0.0	13.8	7.5
Other (e.g., medicine)	27.3	1.2	5.0

**Table 2:** Professional representation in study sample: Time 1 participants

Physicians	%	Nurses	%	Professional	%
Staff Physician	90.9	Adv Prac Nurse	6.2	Dietitian	14.6
Resident	9.1	Charge Nurse	7.4	Occ Therapist	12.2
TOTAL	100.0	R.N.	75.3	Pharmacist	24.4
		R.P.N.	9.9	Phys Therapist	24.4
		Other	1.2	Social Worker	12.2
		TOTAL	100.0	Speech Lang Path	4.9
				Other	7.3
				TOTAL	100.0

### Unconditional means model of IPC and attitude changes

The first model was a two-level unconditional means model with random intercept. This model estimates the grand mean of scale scores across all individuals and all three survey occasions. It also estimates stability of scores across the total 6-week measurement period, as shown in Equation 1 in the appendix. When written as a composite model, the equations contribute the scale grand mean across individuals and occasions (*γ*_00 _), person-specific means (*ζ*_0__*i*_), and within-person deviations (*ε*_*ij*_) from person-specific means.

The intraclass correlation (ICC) expresses stability of scale scores as the magnitude of between-person to within-person variation. The variance components from Equation 1 provide the between-person variance which is divided by the total variance, (). This is the proportion of total outcome variance that is 'between' persons. Scale results for grand mean scores and ICCs appear in Table 3. For IPC scales of communication, accommodation, and isolation, results are in ascending order according to ICC values for each rater/target group combination. The NWI-NPRS was given to nurses to evaluate physicians; one row is required in Table 3. ATHCTS results for team value, team efficiency, and shared leadership are in alphabetical order.

**Table 3:** IPC and nurse-physician relations behavior, and attitudes to teamwork: Unconditional means model results

**Scale Outcome (rater → target**^**a**^**)**	Grand Mean	ICC (variation that is between persons)
**Communication **(max = 20)		
P→A	13.2	0.27
P→N	13.6	0.50
N→A	13.8	0.54
A→P	12.2	0.56
A→N	13.9	0.61
N→P	12.6	0.71
**Accommodation **(max = 20)		
P→N	14.9	0.07
N→A	14.0	0.50
P→A	14.7	0.53
A→N	14.4	0.54
N→P	12.7	0.57
A→P	13.2	0.69
**Isolation **(max = 12)		
P→A	8.9	0.40
P→N	9.0	0.41
N→P	7.1	0.52
N→A	8.4	0.56
A→P	7.2	0.67
A→N	8.3	0.70
**NWI**-**NPRS**: (N → P; max = 12)	8.3	0.61
**Team Value**^**b **^(max = 66)		
All	55.5	0.70
Allied	56.9	0.70
Nurses	55.4	0.69
Physicians	51.6	0.64
**Team Efficiency**^**b **^(max = 30)		
All	22.3	0.59
Allied	23.3	0.49
Nurses	22.0	0.62
Physicians	21.1	0.54
**Shared Leadership**^**b **^(max = 30)		
All	18.3	0.70
Allied	18.3	0.73
Nurses	18.7	0.69
Physicians	15.5	0.44

^a ^N = nurse, A = allied, P = physician; ^b^For Team Value, Team Efficiency, and Shared Leadership: 'respondents', not 'raters'

### Grand mean scores

Maximum possible scores for the communication and accommodation IPC subcales were both 20. Modeled mean scores were slightly higher for accommodation than communication. Physicians gave higher scores to other groups on IPC scales, and physicians received lower ratings from other groups. Both nurses and allied professionals judged physicians lowest of their two target groups. The IPC isolation subscale and the NWI-NPRS had identical maximum possible scores (12). On the NWI-NPRS, the mean rating nurses gave to physicians was 8.3; this was slightly higher than the 7.1 mean that nurses gave to physicians on the IPC isolation subscale.

Physicians had the lowest grand mean scores of all groups on the ATHCTS subscales. Maximum possible scores for team efficiency and shared leadership were both 30; modeled grand mean scores were lower for all groups on shared leadership than team efficiency.

### Intraclass correlation coefficient

The ICC values for all subscales, rater/target combinations, and target groups, were usually greater than .50 and often approached or exceeded .70 (70% variance between persons, 30% within persons). For most rater/target combinations, over half of the overall scale variance was at the between-person level. Less than half of variance in other-directed IPC ratings and attitudinal self reports was at the within-person level over time, suggesting that individuals differed about their usual levels somewhat less than they differed from each other. The exception was with physician raters, who displayed more within-person variation than either nurses or allied professionals. The difference can be seen in physicians' ratings of both target groups on the IPC communication and isolation subscales where ICCs were .27 and .50 for communication and .40 and .41 for isolation, and in physicians' ratings of nurses' accommodation, where ICC was .07. In contrast, for the IPC subscales, highest ICC values existed for physician targets of the other two rater groups, and for nurses as targets of allied professionals. Physicians also had the lowest ICC self-reports of endorsing shared leadership, at .44, indicating that physicians differed around their mean overall level slightly more than they differed from each other. However the variance about the overall level for physicians was greater than the variances for other groups, which were closer to 30% (ICCs of .73 and .69 respectively).

### Unconditional growth and reliability of IPC and attitude changes

The second series of models included a single predictor, time. Known as an unconditional growth model, this model says that individual *i*'s observed scale score on occasion *j*, *Y*_*ij*_, deviates by ε_*ij *_from his or her true change trajectory over the three survey occasions. The model also estimates inter-individual variation in the rates of change (τ_11_). The full two-level model is given in Equation 2 (see appendix). Residual variances from the level-1 and level-2 models were used to estimate two key quantities--the amount of within-person variation 'explained' by the addition of time as a level-one predictor, and the correlation between initial status (before simulation training) and change over time (after simulation training).

Variance explained by time is a pseudo-*R*^2 ^statistic calculated as shown in Equation 3 in the appendix. The population covariance of the level-2 residuals from Equation 2, , quantifies the covariance between true initial status and true change on IPC and attitudes scores. It can be re-expressed as a correlation coefficient by dividing it by the square root of the product of its variance components, i.e., . This is the population correlation between true initial status and true change over three survey occasions.

Results for rater-target combinations and respondent groups are given in Table 4, displayed in ascending order according to within-person variance explained by time (pseudo-*R*^2 ^for level-1) for each scale/subscale. Within-person variance explained by time was low for the majority of rater-target combinations on IPC subscales and the NWI, and for ATHCTS subscales. Apart from the value for physicians judging nurses on IPC accommodation, which was .51, variance explained was usually not greater than .20. Some *R*^2 ^values for the unconditional growth model were negative. This occurs when a model's level-1 predictor increases the within-person residual variance, [29,30].

**Table 4:** IPC and nurse-physician relations behavior, and attitudes to teamwork: Unconditional growth model results

**Scale Outcome (rater → target**^**a**^**)**	Variance explained by time	Correlation: initial status and true change
**Communication**		
P→A	.03	--^c^
P→N	.04	--^c^
A→N	.09	-.60
N→P	.17	-.34
A→P	.21	-.48
N→A	.22	-.54*
**Accommodation**		
P→A	-.10	--^c^
A→N	-.02	--^c^
N→P	.00	-.15
N→A	.06	.03
A→P	.08	.28
P→N	.51	--^c^
**Isolation**		
P→N	-.08	--^c^
P→A	-.07	--^c^
A→N	.03	-.47
N→A	.08	-.47
N→P	.11	-.78*
A→P	.11	-1.00*
**NWI**-**NPRS**: (N → P)	.01	--^c^
**Team Value**^**b**^		
Physicians	-.02	--^c^
All	.09	.02
Allied	.13	.91*
Nurses	.19	-.21
**Team Efficiency**^**b**^		
Nurses	.00	--^c^
All	.03	-.43
Physicians	.11	--^c^
Allied	.19	-.62
**Shared Leadership**^**b**^		
Nurses	.07	-.71
All	.11	-.58*
Physicians	.11	--^c^
Allied	.20	-.34

^a ^N = nurse, A = allied, P = physician; ^b^For Team Value, Team Efficiency, and Shared Leadership: 'respondents', not 'raters'; ^c ^Non-estimable (not positive definite); *P < .05

Correlations between initial status and change are given in the third column of Table 4. Most correlations that could be estimated were negative. Four were statistically significantly different from zero and large (<-.50). Negative correlations indicate that respondents who reported lower IPC, NWI, and ATHCTS scores prior to simulation training gave higher scores at later survey occasions than respondents with higher initial scores. Correlations were very large for physician targets on IPC isolation with nurses and allied professionals as raters (-.78, -1.0). In other words, those originally rating physicians as most isolated (giving low scores) reported the largest (positive) ratings-changes over time. The other statistically significant negative relationships between initial status and change were found for nurses rating allied professionals (-.54), and for all combined respondents on the shared leadership subscale of the ATHCTS (-.58). The lone statistically significant positive relationship was for allied professionals' responses to the team value scale (.91). For allied professionals, gains in perceived team value were most likely among those with higher initial perceptions of team value.

In many instances the correlation could not be estimated. This was always because the variance component for the rate of change () could not be calculated. This result occurred frequently with physician raters and was probably a function of too few observations available for analysis.

Reliability of outcome measures was estimated with Cronbach's alpha for each survey occasion. We used a structural equation modeling (SEM) framework [31] and Mplus 5.2 [32] to derive 95% confidence intervals and test the equality of alpha at adjacent survey occasions for nurses and allied health professionals. Alpha could not be estimated for physician outcomes in the SEM setup because models would not converge, likely because of small sample sizes. In these instances SAS PROC CORR was used.

Alpha estimates are shown for the three survey occasions in Table 5. Reliability of shared leadership was moderate among nurses and allied health professionals and usually low for physicians. Confidence intervals were wide, but alpha estimates were not statistically different. Alpha was lower for team efficiency and poor for physicians. Alpha for team value was high for all groups. Confidence intervals were narrow. For nurses and allied health professionals, alpha increased from one period to the next and the differences were statistically significant.

**Table 5:** ATHCTS reliabilities, by time and profession

		Respondents		
		
Scale	Time	Physicians	Nurses	Allied Health Professionals
Shared Leadership	1	.72^a^	.70 (.59, .82)^c^	.67 (.44, .90)
	2	.35^a ^(.60)^b^	.73 (.63, .83)	.60 .(42, .79)
	3	.05^a ^(.70)^b^	.62 (.46, .77)	.70 (.48, .92)
	
	1/2	.72, .35	.70, .73	.67, .60
		no conv^d^	n.s.	n.s.
	
	2/3	.35, .05	.73, .62	.56, .70
		no conv	n.s.	n.s.

Team Efficiency	1	.31	.57 (.42, .73)	.64 (.41, .89)
	2	.06^a^	.63 (.49, .76)	.61 (.48, .80)
	3	.47^a^	.65 (.51, .78)	.56 (.29, .84)
	
	1/2	.31, .06	.57, .63	.64, .61
		no conv	n.s.	n.s.
	
	2/3	.06, .47	.65, .65	.66, .56
		no conv	n.s.	n.s.

Team Value	1	.90^a^	.86 (.81, .91)	.79 (.69, .89)
	2	.95^a^	.95 (.92, .99)	.93 (.85, 1.00)
	3	.87^a^	.99 (.96, 1.00)	.97 (.93, 1.00)
	
	1/2	.92, 1.01	.86, .95	.79, .93
		no conv	**	*
	
	2/3	.93, .99	.90, .99	.85, .97
		no conv	**	*

^a^PROC CORR; ^b^alpha if 1 item deleted; ^c^Asymptotic distribution-free CIs; ^d^No convergence *P < .05, **P < .01; n.s. = not significant

### Linear growth with a person-level covariate

After fitting the unconditional growth model, we investigated growth models for ATHCTS subscales as outcome measures, specifying variation in intercepts and slopes to be related to the covariate, leadership capacity. We focused on the ATHCTS because an association with attitudes was considered more likely than with judgments of others' interprofessional collaboration. The two-level growth model is shown in Equation 4 of the appendix. Equation 4 stipulates that attitude scores are a function of a level-1 intercept that varies as a function of leadership capacity, a within-person residual, and an interaction between leadership capacity and time. The latter is a cross-level interaction to test for differential change with respect to the covariate. Equation 4 also contains a random component for the intercept, , permitting intercepts (initial-status attitudes scores) to vary across people. The level-2 equation predicting level-1 time and the time × leadership interaction, *π*_1__*i *_= *γ*_10 _+*γ*_11 _(Leadership) _*j*_, omits the random effect for time that was present in the model for unconditional growth (Equation 2) because experimentation revealed non-convergent models which indicated that including the random effect would over-complicate the model.

The model in Equation 4 was estimated for the three ATHCTS subscales. Time was entered as a continuous variable to produce the linear growth model. These models yielded coefficients relating to effects of time, leadership, and the time-by-leadership interaction. Among models estimated separately for each subscale and profession, only one coefficient for time was statistically different from zero at *P *< .05 (physicians, shared leadership subscale). Therefore we tentatively concluded that attitudes toward health care teamwork likely did not exhibit linear growth in the six-week follow-up.

The Equation 4 model was re-estimated with time specified as a series of dummy variables. Fixed effects parameter estimates for the three ATHCTS subscales and three professions are reported in Additional File 2: Fixed Effects. We do not discuss the results in depth here, except to note the inconsistent effect of including leadership capacity on pseudo-*R*^2 ^values for differences in initial status. When compared with the unconditional growth model, adding leadership capacity as a predictor had more relevance for explaining variance in initial status on the shared leadership subscale than any other. The corollary of this result is taken up in Table 6, which reports statistically significant mean score differences on pairwise comparisons of leadership capacity for ATHCTS subscales and survey occasions. Least squares mean scores are given.

**Table 6:** Leadership capacity: statistically significant differences of mean scores, by time, ATHCTS subscale, and profession

Comparison Values of Leadership Capacity	Time 1	Time 2	Time 3
**Shared Leadership**			
Physicians (N = 11)			
1 0	15.3, 27.0 *	13.2, 20.0	14.7, 17.0
2 0	18.5, 27.0 *	14.2, 20.0	16.2, 17.0
Nurses (N = 83)			
1 0	18.2, 21.6 *	17.5, 20.2	18.4, 20.2
2 0	18.1, 21.6 *	18.2, 20.2	18.7, 20.2
3 1	23.0, 18.2 *	22.7, 17.5 *	22.4, 18.4
3 2	23.0, 18.0 *	22.7, 18.3	22.4, 18.7
Allied Health Professionals (N = 42)			
2 0	21.0, 16.9 *	19.6, 16.8	18.7, 16.3
3 0	30.0, 16.9 *	25.0, 16.8 *	26.0, 16.3 *
3 1	30.0, 18.2 *	25.0, 18.2	26.0, 18.3 *
3 2	30.0, 21.0 *	25.0, 19.6	26.0, 18.7
**Team Efficiency**			
Nurses (N = 83)			
2 0	21.2, 22.9	20.1, 23.3 *	21.5, 22.4
2 1	21.2, 22.1	20.1, 22.4 *	21.5, 21.6
3 2	24.5, 21.2	25.4, 20.1 *	25.0, 21.5
**Team Value**			
Nurses (N = 83)			
3 2	58.8, 50.9 *	54.8, 53.2	56.6, 51.5

*P < .05

Attitudes toward shared leadership had the largest number of significant differences. Initial differences were observed for all health professional groups and were most apparent for allied health professionals. Two patterns are evident. First, groups with the maximum value on leadership capacity (3) always reported higher endorsement of shared leadership than comparison groups with leadership capacity values <3. These differences were large among allied health professionals. Second, physicians and nurses with leadership capacity values of zero had higher shared leadership scores than those with greater leadership capacity values. The same general pattern of results was found for attitudes toward team efficiency and attitudes toward team value, although there were many fewer statistically significant differences.

Very few pre-training score differences were sustained over the full post-simulation follow-up period. One exception occurred with shared leadership where a few initial differences on leadership capacity involving nurses and allied health professionals were found again at the two-week follow-up, the six-week follow-up, or all three occasions.

Attitudes toward shared leadership can be examined informally between professions. The group that had strongest endorsement of shared leadership was allied health professionals. Nurses had an intermediate position between allied professionals and physicians. Low-scoring physicians with leadership values of 1 and 2 reported scores approximately only half as large as physicians reporting higher endorsement of shared leadership whose leadership capacity values were zero.

## Discussion

The objective of this study was to investigate short-term associations between simulation training and health professionals' judgments of IPC by other groups, and self-reports of attitudes toward working in health care teams. Simple multilevel models examined initial health professional group differences before training and between- and within-person change shortly after.

On IPC factors, physicians gave higher ratings to nurses and allied health professionals than they received from those groups, a finding consistent with previous studies [33-35]. Both nurses and allied health professionals rated physicians lowest of the target groups. Furthermore, physicians always had the lowest self-reported scores on attitudes about the value of teams, team efficiency, and shared team leadership. Shared leadership had the least support of any of the three constructs, and physicians endorsed it the least. Because of the pivotal role of physicians in Canadian health care [3], this should concern health care managers who may wish to implement interprofessional care models for patient care delivery.

The primary questions relating to simulation training and the multilevel model concern stability and change, that is, inter-individual differences in change over time. Variation in IPC and attitudes scores was largely explainable by characteristics differing between persons instead of those that changed within persons over time. The situation with physicians was somewhat different for their IPC scale ratings of others. For physicians there was slightly (or substantially) less between-person variation than within-person variation. This finding could be partially explained by the very small number of physicians in the study (N = 14) and consequent greater physician homogeneity.

The models for unconditional growth typically revealed negative correlations between IPC and attitude levels before simulation training, and subsequent change, but few correlations were statistically significant. Lower scores on the isolation subscale represented qualitatively worse assessments of IPC-related isolation--more isolation--while higher scores represented less isolation. The two correlations for ratings of physicians by nurses and allied professionals on isolation were large and negative. The negative and statistically significant correlations for ratings of physicians suggest that initial assessments of high isolation (low scale scores) were followed by positive change--less isolation (high scores) post-simulation. On this particular facet, physicians may have been indirect beneficiaries of nurses' simulation training because the trend from pre- to post-simulation training was for nurses to perceive physicians as being less isolated over time, albeit a brief time. Nurses may have acquired better understanding of the physicians' role and perspective.

Very little within-person variation in IPC and attitudes scores was attributable to time. This is understandable in light of the brief time that passed between the pre-simulation survey occasion and the final occasion six weeks later. Studies with longer follow-up periods may yield different results.

Leadership capacity was derived from job characteristics and entered as a predictor to multilevel models for professions and pairwise comparisons of leadership capacity. The results suggest the existence of an initial, pre-simulation 'bump' in reported support for teamwork. For most professions and leadership capacity comparisons where there were initial differences, the differences were not sustained two and six weeks after training. In one comparison involving allied health professionals the difference was observed at all three survey occasions, although a score decline was still apparent even for the high-leadership-capacity individuals.

The comparison of mean scores for different levels of leadership capacity suggests that two 'classes' of leadership capacity groups exist among the study participants. As one might anticipate, higher endorsement of shared leadership occurred among nurses and allied health care professionals who had at least six years of post-licensure experience, managerial obligations, and no direct patient care tasks. These individuals placed higher value on shared leadership prior to IPC simulation training and may have maintained it through the third survey occasion. A second group--concentrated among physicians and nurses who had less than six years experience, no managerial responsibilities, and direct patient care tasks--expressed greater support for shared leadership and team efficiency as well. Although the latter group stands in contrast to senior colleagues, its support for shared leadership was not sustained. For these physicians and nurses, continuing institutional or group support may help sustain endorsement of interprofessional care. The importance of shared leadership for organizational change has been discussed and evaluated [22]. Reeves et al. [20] integrated it into a model of interprofessional teamwork using terms such as professional and organizational power; the concepts are related in that health professionals who feel empowered to influence their work processes and improve their work conditions are more likely to engage in change [[20]:p59].

The study found supportive teamwork attitudes in predictable and unconventional places: among participants with and without current high-leadership roles, respectively. Some may see these individuals as formal and informal leaders [36] or leverage repositories for future developments in interprofessional care. However, the lack of sustained change among younger participants suggests that continuing socialization towards shared leadership may be necessary [3].

### Limitations

The study had several limitations. First, the measurement occasions were compressed into a short post-simulation period of six weeks. It may not have been reasonable to expect to be able to model linear growth of IPC assessments and teamwork attitudes in this time. Second, professional representation in subgroups was unbalanced. There were many more nurse participants than others, and very few physicians. Generalizability may be limited. Third, measurement equivalence of the survey instruments over time is unknown and may not exist. This problem is not unique to our study. Lack of knowledge of scale equivalence of repeated measures is a long-standing concern in longitudinal research. Finally, the study design cannot support strong inferences about the causal role of simulation training in changing IPC assessment and attitudes over time. While temporal orderings between leadership capacity, simulation training, and outcomes are clear, the study did not employ a control/comparison group.

## Conclusions

For simulation scholars we believe these results may undermine confidence in the ability of simulation activities to substantially improve interprofessional attitudes in the long run. Perceptions held by other health professionals and by physicians themselves concerning physicians' interprofessional effectiveness may be difficult to influence in a positive direction if simulation training initiatives are only used intermittently and without strong institutional support. Perceptions of physicians' collaborative behaviors in areas of communication, accommodation and shared leadership are areas of particular concern. The high initial baseline endorsement of shared leadership among less-experienced physicians and nurses and more-experienced health professionals may be reason for optimism, but it should be recognized that this support declined and post-training 'recovery' of support for shared leadership was very modest.

While our position appears contrary to the majority of studies concluding that simulation is an effective IPE teaching tool, our study's novel characteristics distinguish it from others. For example, the participants were active clinicians and not students. The focus of training was interprofessional relationships rather than skill-based practices, such as resuscitation and emergency interventions. Outcomes constructs were measured very soon after training with instruments whose psychometric properties are known.

Attitudes highly supportive of shared team leadership may be a lever that IPE and interprofessional simulation interventionists--and health care institutions more broadly--can use to engage clinicians in interprofessional care. Simulation training programs that are designed to promote shared leadership within interprofessional training groups should be planned and evaluated. Finally, health care institutions should sanction interprofessional education more widely to support teamwork attitudes among their actively practicing advocates and proponents.

## Appendix

Equation 1:(1)

Equation 2:(2)

Equation 3:(3)

Equation 4:(4)

## Competing interests

The authors declare that they have no competing interests.

## Authors' contributions

CK participated in choosing outcomes, measurement instruments, and analysis methods; performed statistical analysis and interpreted output; and drafted the methods and results sections. KM participated in choosing outcomes and analysis methods; drafted the article's background section; and revised the final draft for important content, discussion, and conclusion material. MvS participated in the design of simulation training and recruiting participants; selected measurement instruments; conducted simulation training sessions; drafted the paper's background section; and revised the final draft for important content, discussion, and conclusion material. SR participated in selecting outcomes; drafted the background section; and revised the final draft for important content, discussion, and conclusion material. All authors read and approved the final version of the manuscript.

## Authors' Information

CK: Research Coordinator, Li Ka Shing Knowledge Institute of St. Michael's Hospital, Toronto, Ontario, Canada. KM: Dean, School of Health Sciences, Humber Institute of Technology & Advanced Learning, Toronto, Ontario, Canada. MvS: Director, Canadian Health Care Innovations, Guelph, Ontario, Canada. SR: Scientist, Li Ka Shing Knowledge Institute of St. Michael's Hospital, Toronto, Canada; *and *Director of Research, Centre for Faculty Development, Li Ka Shing International Healthcare Education Centre, Toronto, Canada; *and *Scientist, Wilson Centre for Research in Education, University of Toronto, Canada; *and *Associate Professor, Department of Psychiatry, University of Toronto, Canada.

## Pre-publication history

The pre-publication history for this paper can be accessed here:

http://www.biomedcentral.com/1741-7015/9/29/prepub

## Supplementary Material

Additional file 1 **The Gift**. Example scenario given to all participants; designed to promote shared decision making and understanding of multiple perspectives. The roles were assigned to participants who did not work in the focal profession/position. For example, a nurse would be given a social worker role. Within workshop groups, roles were assigned only if the profession was represented among the participants. Click here for file

Additional file 2 **Fixed Effects**. Shared Leadership: fixed effects estimates for time and leadership capacity, 3 professions. Click here for file

## References

[B1] Miller KL Reeves S Zwarenstein M Beales JD Kenaszchuk C Conn LG Nursing emotion work and interprofessional collaboration in general internal medicine wards: a qualitative study J Adv Nurs 2008 64 332 343 10.1111/j.1365-2648.2008.04768.x 18990112

[B2] Reeves S Rice K Conn LG Miller KL Kenaszchuk C Zwarenstein M Interprofessional interaction, negotiation and non-negotiation on general internal medicine wards J Interprof Care 2009 23 633 645 10.3109/13561820902886295 19842957

[B3] Reeves S MacMillan K van Soeren M Leadership of interprofessional health and social care teams: a socio-historical analysis J Nurs Man 2010 18 258 264 10.1111/j.1365-2834.2010.01077.x 20546465

[B4] Oandasan IF The way we do things around here: advancing an interprofessional care culture within primary care Can Fam Physician 2009 55 1173 1174 20008587 PMC2793211

[B5] Velji K Baker GR Fancott C Andreoli A Boaro N Tardif G Aimone E Sinclair L Effectiveness of an adapted SBAR communication tool for a rehabilitation setting Healthc Q 2008 11 72 79 18382165 10.12927/hcq.2008.19653

[B6] Zwarenstein M Reeves S Knowledge translation and interprofessional collaboration: where the rubber of evidence-based care hits the road of teamwork J Contin Educ Health Prof 2006 26 46 54 10.1002/chp.50 16557506

[B7] Hogg W Lemelin J Dahrouge S Liddy C Armstrong CD Legault F Dalziel B Zhang W Randomized controlled trial of anticipatory and preventive multidisciplinary team care Can Fam Physician 2009 55 e76 e85 20008582 PMC2793206

[B8] Barrett J Curran V Glynn L Godwin M CHSRF synthesis: Interprofessional Collaboration and Quality Primary Healthcare 2007 Ottawa: Canadian Health Services Research Foundation http://www.chsrf.ca/Migrated/PDF/ResearchReports/CommissionedResearch/SynthesisReport_E_rev4_FINAL.pdf

[B9] Canadian Interprofessional Health Collaborative A National Interprofessional Competency Framework 2010 Vancouver: University of British Columbia

[B10] Hammick M Freeth D Koppel I Reeves S Barr H A best evidence systematic review of interprofessional education: BEME Guide no. 9 Med Educ 2007 29 735 751 10.1080/01421590701682576 18236271

[B11] Reeves S Zwarenstein M Goldman J Barr H Freeth D Koppel I Hammick M Interprofessional Education: Effects on Professional Practice and Health Care Outcomes 2008 1 London: John Wiley & Sons (Cochrane Review). Cochrane Library 10.1002/14651858.CD002213.pub2 18254002

[B12] Salas E Burke CS Cannon-Bowers JA Kraiger K What we know about designing and delivering team training: tips and guidelines Creating, Implementing, and Managing Effective Training and Development: State-of-the-Art Lessons for Practice 2002 San Francisco: Jossey-Bass 234 261

[B13] Gaba DM What does simulation add to teamwork training? 2006 http://www.webmm.ahrq.gov/perspective.aspx?perspectiveID=20 Morbidity and Mortality Rounds on the Web. AHRQ

[B14] Schmitz CC Chipman JG Luxenberg MG Beilman GJ Professionalism and communication in the intensive care unit: reliability and validity of a simulated family conference Simul Healthc 2008 3 224 238 10.1097/SIH.0b013e31817e6149 19088667

[B15] Cameron A Rennie S DiProspero L Langlois S Wagner S Potvin M Dematteo D LeBlanc V Reeves S An introduction to teamwork: findings from an evaluation of an interprofessional education experience for 1000 first-year health science students J Allied Health 2009 38 220 226 20011821

[B16] Raupach T Muenscher C Anders S Steinbach R Pukrop T Hege I Tullius M Web-based collaborative training of clinical reasoning: a randomized trial Med Teach 2009 31 e431 e437 10.1080/01421590903095502 19811180

[B17] Zeltser MV Nash DB Approaching the evidence basis for aviation-derived teamwork training in medicine Am J Med Qual 2010 25 13 23 10.1177/1062860609345664 19801422

[B18] Issenberg SB McGaghie WC Petrusa ER Lee GD Scalese RJ Features and uses of high-fidelity medical simulations that lead to effective learning: a BEME systematic review Med Teach 2005 27 10 28 10.1080/01421590500046924 16147767

[B19] Martin V Rogers A Leading Interprofessional Teams in Health and Social Care 2004 London: Routledge

[B20] Reeves S Lewin S Espin S Zwarenstein M Interprofessional Teamwork for Health and Social Care 2010 Oxford: Wiley-Blackwell

[B21] Caplan G Caplan RB Erchul WP A contemporary view of mental health consultation: comments on 'Types of Mental Health Consultation' by Gerald Caplan (1963) J Educ Psychol Consul 1995 6 23 30 10.1207/s1532768xjepc0601_2

[B22] Porter-O'Grady T Researching shared governance: a futility of focus J Nurs Adm 2003 33 251 252 12690257 10.1097/00005110-200304000-00011

[B23] Adams A Bond S Arber S Development and validation of scales to measure organisational features of acute hospital wards Int J Nurs Stud 1995 32 612 627 10.1016/0020-7489(95)00041-1 8926161

[B24] Kenaszchuk C Reeves S Nicholas D Zwarenstein M Validity and reliability of a multiple-group measurement scale for interprofessional collaboration BMC Health Serv Res 2010 10 83 10.1186/1472-6963-10-83 20353577 PMC2867963

[B25] Aiken LH Patrician PA Measuring organizational traits of hospitals: the Revised Nursing Work Index Nurs Res 2000 49 146 153 10.1097/00006199-200005000-00006 10882319

[B26] Heinemann GD Schmitt MH Farell MP Brallier SA Development of an attitudes toward health care teams scale Eval Health Prof 1999 22 123 142 10.1177/01632789922034202 10350960

[B27] Singer JD Willett JB Applied Longitudinal Data Analysis: Modeling Change and Event Occurrence 2003 New York: Oxford University Press

[B28] Littell RC Milliken GA Stroup WW Wolfinger RD SAS System for Mixed Models 1996 Cary, NC: SAS Institute

[B29] Kreft I de Leeuw J Introducing Multilevel Modeling 1998 Thousand Oaks, CA: Sage

[B30] Snijders TAB Bosker RJ Multilevel Analysis: An Introduction to Basic and Advanced Multilevel Modeling 1999 Thousand Oaks, CA: Sage

[B31] Maydeu-Olivares A Coffman DL Garcia-Forero C Gallardo-Pujol D Hypothesis testing for coefficient alpha: an SEM approach Behav Res Methods 2010 42 618 625 10.3758/BRM.42.2.618 20479193 PMC3027072

[B32] Muthén L Muthén B Mplus User's Guide 1998 Fifth Los Angeles, CA: Muthén & Muthén;

[B33] Verschuren PJ Masselink H Role concepts and expectations of physicians and nurses in hospitals Soc Sci Med 1997 45 1135 1138 10.1016/S0277-9536(97)00043-9 9257405

[B34] Thomas EJ Sexton JB Helmreich RL Discrepant attitudes about teamwork among critical care nurses and physicians Crit Care Med 2003 31 956 959 10.1097/01.CCM.0000056183.89175.76 12627011

[B35] Krogstad U Hofoss D Hjortdahl P Doctor and nurse perception of inter-professional co-operation in hospitals Int J Qual Health Care 2004 16 497 507 10.1093/intqhc/mzh082 15557359

[B36] Østergaard HT Østergaard D Lippert A Implementation of team training in medical education in Denmark Qual Saf Health Care 2004 13 i91 i95 15465962 10.1136/qshc.2004.009985 PMC1765784

