# Supplementary material for: Interprofessional simulated learning: short-term associations between simulation and interprofessional collaboration
Source: BMC Med. 2011 Mar 28;9:29. doi: 10.1186/1741-7015-9-29 (PMC3224569; doi:10.1186/1741-7015-9-29)
Supplement: Additional file 1 — The Gift. Example scenario given to all participants; designed to promote shared decision making and understanding of multiple perspectives. The roles were assigned to participants who did not work in the focal profession/position. For example, a nurse would be given a social worker role. Within workshop groups, roles were assigned only if the profession was represented among the participants. [file 1741-7015-9-29-S1.DOC]

# Additional figure 1 Example scenario given to all participants; designed to promote shared decision making and understanding of multiple perspectives. The roles were assigned to participants who did not work in the focal profession/position. For example, a nurse would be given a social worker role. Within workshop groups, roles were assigned only if the profession was represented among the participants.

# Simulation scenario given to all participants: The Gift

A former patient from the Cardiac Health System has donated a substantial gift of $250,000. Mr. James was well known in the cardiac system and received care from many members of the team throughout his prolonged length of stay. He had an elective angiogram and remained in hospital for immediate surgery due to a severe blockage in his left main coronary artery. Unfortunately, Mr. James had a stroke during the surgery and was left with some deficits that required input from the whole team including the Social Worker, Speech Language Pathologist, Physiotherapist, Occupational Therapist, Dietitian, and the Nurse/Physician team. The Director of the Foundation has called this meeting and therefore will be facilitating. She has said that this patient specifically wanted the money spent in the Cardiac program. Today you are getting together as a team to talk about how the money could be spent. There is some time pressure as the final decision needs to be made today. His two daughters will be attending the meeting. One daughter is a nurse; she has some interest in how the money will be spent. The second daughter has been very quiet, and the family does not seem to be in agreement about the donation.

### Roles given to participants

**Clinical Leader, Cardiology**

You would like to see the money spent on renovating the staff lounge and buying new furniture, microwave, TV and fridge. You feel your role is to support the staff and they are constantly talking about the state of the lounge. You feel it would have a huge impact on morale, which is very low at the moment. The staff turnover in your unit is about 15% and the costs are really crippling as well as the overtime it is causing. Your director is pressuring you to meet your budget and you do not see being able to do it without improving morale.

**Professional Practice Leader representing Allied Health**

You would like to see the money go toward renovation of the rehab gym. You really aren’t sure why you were invited to the meeting. You find yourself involved in many meetings in this role that appear to waste time. You have a clinic waiting now and are anxious to get back. In addition you always feel like other disciplines get all the money and that the invitation was only a token to make it appear like you were involved.

**Director of the Foundation [chair of the meeting]**

All of the team members have different ideas and need to reach a consensus on the best way to spend the money. If you do not come to a consensus soon, the donor will back out. You are aware that the family members who are attending the meeting had some concerns about the way their family received care during their father’s illness, and have some ideas (not shared with their father) about how the money should be spent. Due to time constraints, only one meeting is permitted to agree on how the money will be spent.

**Older Daughter**

You are anxious to see the money put to good use. As a retired nurse (retired 2 years ago after 35 years of practice) you think families should have more to do with the care of a loved one. You found the care your father received at the hospital adequate but there were many times when you felt your concerns were dismissed and there was no one there to help you understand what was going on. You want the money to go to something that will help families get better information about the patient and have more contact with the team. You are volunteering at a senior’s centre where they are teaching seniors to use the Internet and wonder if there is something in that for a solution to communication problems.

**Younger Daughter**

You are angry that your sister has managed to convince your dad to donate a huge chunk of money to the hospital. You think that the money is yours and that your inheritance is being squandered because hospitals are notorious for inappropriate spending. Besides, your sister is the nurse and so no one ever listens to you.

**Physician Leader, Chief of Cardiology**

You would like to see the money put toward a new table and fluoroscopy equipment for the cardiac catheterization lab. You came to the hospital with the promise of new equipment and this has not materialized even with recent renovations. You are quite rushed and don’t think you need to spend a lot of time in this meeting.

**Social Worker**

You have been involved with the family for some time, you think that you know them very well and you see yourself as the only one to assist with the disbursement of the funds. You would like to see the money spent on creating a new patient and family-centered waiting room for visitors on the second floor outside the CCU. There is currently nowhere to meet with families for private discussions. You have a recent personal experience with a family member in CCU and have a strong opinion about the importance of this. You are frustrated that you seem to be the only one who values patient- and family-centered care.

## Clinical Nurse Specialist, Cardiology

Your role has recently changed from a nurse educator to a CNS. Since graduation from the NP program you find yourself dealing with issues not related to cardiology and so you see this as an opportunity to offload the nuisance calls such as skin and wound care to concentrate on cardiology. You heard someone mention that the costs associated with skin breakdown were escalating and so you would like to purchase specialty mattresses that will better manage skin care breakdown.
