# Supplementary material for: Interprofessional simulated learning: short-term associations between simulation and interprofessional collaboration
Source: BMC Med. 2011 Mar 28;9:29. doi: 10.1186/1741-7015-9-29 (PMC3224569; doi:10.1186/1741-7015-9-29)
Supplement: Additional file 2 — Fixed Effects. Shared Leadership: fixed effects estimates for time and leadership capacity, 3 professions. [file 1741-7015-9-29-S2.DOC]

**Additional table 1 Shared Leadership: fixed effects estimates for *time* and *leadership capacity,* 3 professions**

| Effect | Ldr cap^1^ | Time | Physicians | | | |  | Nurses | | | |  | Allied Health Professionals | | | |
| --- | --- | --- | --- | --- | --- | --- | --- | --- | --- | --- | --- | --- | --- | --- | --- | --- |
|  |  |  | Est | SE | t | P |  | Est | SE | t | P |  | Est | SE | t | P |
| Intercept |  |  | 27.00 | 3.27 | 8.27 | <.0001 |  | 21.57 | 1.29 | 16.75 | <.0001 |  | 16.86 | 1.36 | 12.40 | <.0001 |
| Leadership  Capacity | 0 |  | 0.00 | . | . | . |  | 0.00 | . | . | . |  | 0.00 | . | . | . |
|  | 1 |  | -11.75 | 3.46 | -3.39 | 0.01 |  | -3.44 | 1.47 | -2.33 | 0.02 |  | 1.62 | 1.53 | 1.06 | 0.29 |
|  | 2 |  | -8.50 | 4.00 | -2.13 | 0.07 |  | -3.57 | 1.72 | -2.08 | 0.04 |  | 4.14 | 1.92 | 2.15 | 0.04 |
|  | 3 |  | .^2^ | . | . | . |  | 1.43 | 2.35 | 0.61 | 0.55 |  | 13.14 | 3.85 | 3.42 | 0.00 |
| Time |  | 1 | 0.00 | . | . | . |  | 0.00 | . | . | . |  | 0.00 | . | . | . |
|  |  | 2 | -7.00 | 3.43 | -2.04 | 0.07 |  | -1.29 | 1.02 | -1.26 | 0.21 |  | -0.10 | 1.27 | -0.08 | 0.94 |
|  |  | 3 | -10.00 | 3.43 | -2.91 | 0.01 |  | -1.42 | 1.08 | -1.31 | 0.19 |  | -0.54 | 1.36 | -0.40 | 0.69 |
| Leadership capacity x Time | 0 | 1 | 0.00 | . | . | . |  | 0.00 | . | . | . |  | 0.00 | . | . | . |
|  | 0 | 2 | 0.00 | . | . | . |  | 0.00 | . | . | . |  | 0.00 | . | . | . |
|  | 0 | 3 | 0.00 | . | . | . |  | 0.00 | . | . | . |  | 0.00 | . | . | . |
|  | 1 | 1 | 0.00 | . | . | . |  | 0.00 | . | . | . |  | 0.00 | . | . | . |
|  | 1 | 2 | 4.96 | 3.66 | 1.35 | 0.20 |  | 0.67 | 1.18 | 0.57 | 0.57 |  | -0.17 | 1.41 | -0.12 | 0.91 |
|  | 1 | 3 | 9.48 | 3.69 | 2.57 | 0.03 |  | 1.69 | 1.23 | 1.37 | 0.17 |  | 0.38 | 1.49 | 0.26 | 0.80 |
|  | 2 | 1 | 0.00 | . | . | . |  | 0.00 | . | . | . |  | 0.00 | . | . | . |
|  | 2 | 2 | 2.72 | 4.69 | 0.58 | 0.57 |  | 1.58 | 1.40 | 1.13 | 0.26 |  | -1.33 | 1.74 | -0.76 | 0.45 |
|  | 2 | 3 | 7.72 | 4.69 | 1.65 | 0.13 |  | 2.15 | 1.44 | 1.49 | 0.14 |  | -1.75 | 1.81 | -0.97 | 0.34 |
|  | 3 | 1 | . | . | . | . |  | 0.00 | . | . | . |  | 0.00 | . | . | . |
|  | 3 | 2 | . | . | . | . |  | 1.03 | 2.11 | 0.49 | 0.63 |  | -4.90 | 3.42 | -1.43 | 0.16 |
|  | 3 | 3 | . | . | . | . |  | 0.77 | 2.33 | 0.33 | 0.74 |  | -3.46 | 3.45 | -1.00 | 0.32 |
| R^2^ for initial status ($\sigma_{0}^{2}$)^3^ | | | NPD^4^ | | | |  | .20 | | | |  | .49 | | | |
| *Note* ^1^ Leadership capacity ^2^ No physicians with leadership capacity=3  ^3^ Unconditional growth model is the comparison model ^4^ Comparison model for unconditional growth was not positive definite. Est: Estimate SE: Standard error | | | | | | | | | | | | | | | | |

**Additional table 2 Team Efficiency: fixed effects estimates for *time* and *leadership capacity,* 3 professions**

| Effect | Ldr cap^1^ | Time | Physicians | | | |  | Nurses | | | |  | Allied Health Professionals | | | |
| --- | --- | --- | --- | --- | --- | --- | --- | --- | --- | --- | --- | --- | --- | --- | --- | --- |
|  |  |  | Est | SE | t | P |  | Est | SE | t | P |  | Est | SE | t | P |
| Intercept |  |  | 26.00 | 5.19 | 5.01 | 0.00 |  | 22.93 | 1.06 | 21.62 | <.0001 |  | 22.86 | 1.33 | 17.16 | <.0001 |
| Leadership  Capacity | 0 |  | 0.00 | . | . | . |  | 0.00 | . | . | . |  | 0.00 | . | . | . |
|  | 1 |  | -6.38 | 5.50 | -1.16 | 0.28 |  | -0.80 | 1.21 | -0.65 | 0.51 |  | 0.11 | 1.50 | 0.07 | 0.94 |
|  | 2 |  | -4.00 | 6.35 | -0.63 | 0.55 |  | -1.65 | 1.41 | -1.17 | 0.25 |  | 1.43 | 1.88 | 0.76 | 0.45 |
|  | 3 |  | .^2^ | . | . | . |  | 1.57 | 1.94 | 0.81 | 0.42 |  | 3.14 | 3.77 | 0.83 | 0.41 |
| Time |  | 1 | 0.00 | . | . | . |  | 0.00 | . | . | . |  | 0.00 | . | . | . |
|  |  | 2 | -3.00 | 5.06 | -0.59 | 0.57 |  | -0.07 | 0.97 | -0.07 | 0.94 |  | 1.06 | 1.39 | 0.76 | 0.45 |
|  |  | 3 | -1.00 | 5.06 | -0.20 | 0.85 |  | -0.50 | 1.03 | -0.48 | 0.63 |  | -0.29 | 1.49 | -0.20 | 0.84 |
| Leadership capacity x Time | 0 | 1 | 0.00 | . | . | . |  | 0.00 | . | . | . |  | 0.00 | . | . | . |
|  | 0 | 2 | 0.00 | . | . | . |  | 0.00 | . | . | . |  | 0.00 | . | . | . |
|  | 0 | 3 | 0.00 | . | . | . |  | 0.00 | . | . | . |  | 0.00 | . | . | . |
|  | 1 | 1 | 0.00 | . | . | . |  | 0.00 | . | . | . |  | 0.00 | . | . | . |
|  | 1 | 2 | 3.55 | 5.40 | 0.66 | 0.52 |  | 0.37 | 1.12 | 0.33 | 0.74 |  | -0.62 | 1.56 | -0.40 | 0.69 |
|  | 1 | 3 | 3.09 | 5.43 | 0.57 | 0.58 |  | 0.00 | 1.17 | 0.00 | 1.00 |  | 0.51 | 1.64 | 0.31 | 0.76 |
|  | 2 | 1 | 0.00 | . | . | . |  | 0.00 | . | . | . |  | 0.00 | . | . | . |
|  | 2 | 2 | 5.52 | 6.94 | 0.80 | 0.44 |  | -1.12 | 1.32 | -0.84 | 0.40 |  | -2.49 | 1.92 | -1.30 | 0.20 |
|  | 2 | 3 | 2.52 | 6.94 | 0.36 | 0.72 |  | 0.74 | 1.36 | 0.55 | 0.59 |  | -1.42 | 1.99 | -0.71 | 0.48 |
|  | 3 | 1 | . | . | . | . |  | 0.00 | . | . | . |  | 0.00 | . | . | . |
|  | 3 | 2 | . | . | . | . |  | 1.00 | 1.99 | 0.50 | 0.62 |  | 0.94 | 3.77 | 0.25 | 0.80 |
|  | 3 | 3 | . | . | . | . |  | 1.03 | 2.20 | 0.47 | 0.64 |  | 0.29 | 3.80 | 0.08 | 0.94 |
| R^2^ for initial status ($\sigma_{0}^{2}$)^3^ | | | NPD^4^ | | | |  | NPD^4^ | | | |  | .32 | | | |
| *Note* ^1^ Leadership capacity ^2^ No physicians with leadership capacity=3  ^3^ Unconditional growth model is the comparison model ^4^ Comparison model for unconditional growth was not positive definite. Est: Estimate SE: Standard error | | | | | | | | | | | | | | | | |

**Additional table 3 Team Value: fixed effects estimates for *time* and *leadership capacity,* 3 professions**

| Effect | Ldr cap^1^ | Time | Physicians | | | |  | Nurses | | | |  | Allied Health Professionals | | | |
| --- | --- | --- | --- | --- | --- | --- | --- | --- | --- | --- | --- | --- | --- | --- | --- | --- |
|  |  |  | Est | SE | t | P |  | Est | SE | t | P |  | Est | SE | t | P |
| Intercept |  |  | 59.00 | 9.91 | 5.96 | 0.00 |  | 55.79 | 2.14 | 26.04 | <.0001 |  | 57.57 | 2.28 | 25.21 | <.0001 |
| Leadership  Capacity | 0 |  | 0.00 | . | . | . |  | 0.00 | . | . | . |  | 0.00 | . | . | . |
|  | 1 |  | -8.38 | 10.51 | 8.00 | 0.45 |  | -0.85 | 2.45 | -0.35 | 0.73 |  | -0.46 | 2.56 | -0.18 | 0.86 |
|  | 2 |  | -4.00 | 12.13 | 8.00 | 0.75 |  | -4.34 | 2.86 | -1.52 | 0.13 |  | -3.00 | 3.23 | -0.93 | 0.36 |
|  | 3 |  | .^2^ | . | . | . |  | 3.05 | 3.91 | 0.78 | 0.44 |  | 6.43 | 6.46 | 1.00 | 0.33 |
| Time |  | 1 | 0.00 | . | . | . |  | 0.00 | . | . | . |  | 0.00 | . | . | . |
|  |  | 2 | -13.00 | 7.72 | 11.00 | 0.12 |  | 0.86 | 1.71 | 0.50 | 0.62 |  | -1.21 | 1.85 | -0.65 | 0.52 |
|  |  | 3 | -6.00 | 7.72 | 11.00 | 0.45 |  | -1.50 | 1.81 | -0.83 | 0.41 |  | -2.20 | 1.98 | -1.11 | 0.27 |
| Leadership capacity x Time | 0 | 1 | 0.00 | . | . | . |  | 0.00 | . | . | . |  | 0.00 | . | . | . |
|  | 0 | 2 | 0.00 | . | . | . |  | 0.00 | . | . | . |  | 0.00 | . | . | . |
|  | 0 | 3 | 0.00 | . | . | . |  | 0.00 | . | . | . |  | 0.00 | . | . | . |
|  | 1 | 1 | 0.00 | . | . | . |  | 0.00 | . | . | . |  | 0.00 | . | . | . |
|  | 1 | 2 | 13.80 | 8.24 | 1.67 | 0.12 |  | 0.68 | 1.97 | 0.35 | 0.73 |  | 1.21 | 2.06 | 0.59 | 0.56 |
|  | 1 | 3 | 7.02 | 8.30 | 0.85 | 0.42 |  | 1.37 | 2.06 | 0.66 | 0.51 |  | 1.44 | 2.18 | 0.66 | 0.51 |
|  | 2 | 1 | 0.00 | . | . | . |  | 0.00 | . | . | . |  | 0.00 | . | . | . |
|  | 2 | 2 | 13.61 | 10.71 | 1.27 | 0.23 |  | 0.33 | 2.34 | 0.14 | 0.89 |  | 1.78 | 2.54 | 0.70 | 0.49 |
|  | 2 | 3 | 2.61 | 10.71 | 0.24 | 0.81 |  | 1.50 | 2.41 | 0.62 | 0.54 |  | 2.92 | 2.64 | 1.10 | 0.27 |
|  | 3 | 1 | . | . | . | . |  | 0.00 | . | . | . |  | 0.00 | . | . | . |
|  | 3 | 2 | . | . | . | . |  | -4.85 | 3.53 | -1.37 | 0.17 |  | -1.79 | 4.98 | -0.36 | 0.72 |
|  | 3 | 3 | . | . | . | . |  | -0.69 | 3.90 | -0.18 | 0.86 |  | 0.20 | 5.03 | 0.04 | 0.97 |
| R^2^ for initial status ($\sigma_{0}^{2}$)^3^ | | | NPD^4^ | | | |  | .06 | | | |  | -.62 | | | |
| *Note* ^1^ Leadership capacity ^2^ No physicians with leadership capacity=3  ^3^ Unconditional growth model is the comparison model ^4^ Comparison model for unconditional growth was not positive definite. Est: Estimate SE: Standard error | | | | | | | | | | | | | | | | |
